# *In vivo* probing of nascent RNA structures reveals principles of cotranscriptional folding

**DOI:** 10.1093/nar/gkx617

**Published:** 2017-07-14

**Authors:** Danny Incarnato, Edoardo Morandi, Francesca Anselmi, Lisa M. Simon, Giulia Basile, Salvatore Oliviero

**Affiliations:** 1 Dipartimento di Scienze della Vita e Biologia dei Sistemi, Università di Torino, Via Accademia Albertina, 13, Torino, Italy; 2 Italian Institute for Genomic Medicine (IIGM), Via Nizza 52, 10126 Torino, Italy

## Abstract

Defining the *in vivo* folding pathway of cellular RNAs is essential to understand how they reach their final native conformation. We here introduce a novel method, named Structural Probing of Elongating Transcripts (SPET-seq), that permits single-base resolution analysis of transcription intermediates’ secondary structures on a transcriptome-wide scale, enabling base-resolution analysis of the RNA folding events. Our results suggest that cotranscriptional RNA folding *in vivo* is a mixture of cooperative folding events, in which local RNA secondary structure elements are formed as they get transcribed, and non-cooperative events, in which 5′-halves of long-range helices get sequestered into transient non-native interactions until their 3′ counterparts have been transcribed. Together our work provides the first transcriptome-scale overview of RNA cotranscriptional folding in a living organism.

## INTRODUCTION

RNA structure plays a key role in regulating several aspects of the RNA metabolism, like translation, stability and interaction with proteins (1–3). However, to date, how RNA folding takes place *in vivo* has not yet been clarified. There are two main hypotheses on how it can occur. One possibility is that RNA exists after transcription as a random coil, in which any residue within the RNA chain is virtually available for base pairing. Given that the folding space for an RNA molecule is enormous, so that an *n* nucleotides long RNA can assume up to 1.8*^n^* distinct conformations (4), RNA folding in this scenario is likely to be a *non-cooperative* event, characterized by the formation of many low energy non-native structures, formerly known as *kinetic traps* (5,6). This kinetically trapped intermediates are separated from the native conformation by high-energy barriers. *In vitro* RNA folding studies well approximate this condition, as RNA is first heat-denatured, blocked in its denatured conformation by incubation in ice, and then allowed to re-fold by slowly increasing the temperature. Under these conditions, folding of large RNA molecules is a process that can take up to hours before the native functional state is reached.

However, the going assumption for RNA folding is that it occurs in a cotranscriptional fashion. In this scenario, RNA folding is an ordered process in which the 5′ to 3′ directionality of transcription dictates the order of folding events (7). The first *in vitro* study conducted on yeast phenylalanine transfer RNA (tRNA^Phe^) suggested cotranscriptional folding to be a *cooperative* process, in which only correct helical stems are formed during transcription, while no additional or competing helices are produced (8). Oppositely, subsequent studies conducted on MDV-1 RNA, and various *Escherichia coli* RNAs, shown that *in vitro* cotranscriptional folding of these RNA molecules is a *non-cooperative* process. Thus, while transcription took place, non-native secondary structure elements formed, then dissociated at later transcription stages in favor of alternative structures (9,10).

In the last years, many techniques have been proposed to investigate RNA secondary structure *in vivo* on a transcriptome-wide scale. These methods can provide a snapshot of mature RNA conformation through the use of chemical probes able to permeate cell membranes, and to modify unpaired residues within RNA molecules. However, none of them is able to capture the individual steps in RNA folding, thus leaving the open question on how the final RNA conformation is reached.

In trying to address this question, we developed a novel method named Structural Probing of Elongating Transcripts (SPET-seq). This approach, allowed us to capture the structure of individual transcription intermediates at near base-resolution. We applied SPET-seq to *E. coli* cells, and we demonstrated, for the first time, that cotranscriptional RNA folding is a general feature of cellular RNAs, and that it mostly occurs in a cooperative fashion.

## MATERIALS AND METHODS

### Preparation of DNA template for *in vitro* transcription

Genomic DNA was extracted from a 3 ml culture (OD_600_ ∼0.3) of *E. coli* strain DH10B using DNeasy Blood & Tissue Kit (QIAGEN, cat. 69504), following manufacturer instructions. The RNase P gene (rnpB) was amplified by 20 cycles of polymerase chain reaction (PCR) using Phusion^®^ High-Fidelity DNA Polymerase (NEB, cat. M0530S). Oligonucleotides were designed 40-bp upstream of the promoter’s −35 Box to account for the presence of an upstream element, and at the end of the Rho-independent terminator. After PCR, two bands were visible on agarose gel (489- and 601-bp bands), due to the presence of two consecutive terminators with identical sequences. The shorter band (489 bp) was excised, purified, cloned into Zero Blunt^®^ TOPO^®^ vector (Invitrogen, cat. K2880–20) and verified by Sanger sequencing. A total of 60 μg of TOPO-rnpB plasmid were digested using EcoRI, insert was gel purified and used for downstream *in vitro* transcription reaction.

### *In vitro* transcription and DMS modification

*In vitro* transcription reactions were performed in a final volume of 50 μl. Each reaction contained 2 μl *E. coli* RNA Polymerase Holoenzyme (NEB, cat. M0551S), 10 μl *E. coli* RNA polymerase buffer (10×), 1 μl SUPERase• In™ RNase Inhibitor (Ambion, cat. AM2696) and 500 ng template DNA. Reactions were incubated at 37°C for 5 min to allow formation of the RNA Polymerase–DNA binary complex. Transcription was started by addition of 1 μl NTPs (25 mM each), and incubated at 37°C for 10 min. Transcription was stopped by addition of 1 μl DNase I (50 U/μl) and Actinomycin D (Sigma Aldrich, cat. A1410, dissolved in Dimethyl sulfoxide (DMSO) to 5 μg/μl) to a final concentration of 25 ng/μl. Reactions were diluted by addition of 50 μl *E. coli* RNA polymerase buffer 1×. DMS (Sigma Aldrich, cat. D186309) was diluted 1:6 in 100% ethanol to a final concentration of 1.76 M. Diluted Dimethyl sulfate (DMS) was added to reactions to a final concentration of 100 mM. For control samples, an equal volume of a 1:6 dilution of nuclease-free water in 100% ethanol was added. Samples were incubated with moderate shaking (800 RPM) at 25°C for 2 min, after which reactions were immediately transferred to ice. Two volumes of ice-cold RNA binding buffer from RNA Clean & Concentrator™-5 kit (Zymo Research, cat. R1014) supplemented with DTT (Sigma Aldrich, cat. 43815) to a final concentration of 0.7 M, were added to quench DMS and samples were vigorously vortexed for 10 s. RNA was purified on RNA Clean & Concentrator™-5 columns following manufacturer instructions, and eluted in 6 μl of nuclease-free water. A total of 6 μl of 2× RNA Loading Dye (ThermoScientific, cat. R0641) were added to purified RNA. Both DMS-treated and -untreated samples were heated to 95°C for 2 min, and immediately placed on ice. Samples were resolved on a 10% TBE-Urea polyacrylamide gel, and a gel slice corresponding to fragments in the range 50–400 nt was cut. Gel slices were crushed by centrifugation through a punctured 0.5 ml tube, and resuspended in 500 μl of diffusion buffer [500 mM ammonium acetate; 0.05% sodium dodecyl sulphate] supplemented with 60 U SUPERase• In™ RNase Inhibitor, then rotated at 4°C for 16 h to allow passive diffusion of RNA fragments into buffer. RNA was precipitated by addition of 1 ml Isopropanol, and 2 μl Glycogen (20 μg/μl) and resuspended in 6 μl nuclease-free water.

### Bacteria culture and *in vivo* DMS modification

A single colony of *E. coli* strain DH10B was inoculated into 250 ml of LB medium without antibiotics, and grown at 37°C with shaking (150 RPM) for ∼4 h, until OD_600_ was ∼0.3 (log phase). A total of 25 ml aliquots of bacteria were then pelleted by centrifugation at 1800 *g* for 15 min (4°C). After centrifugation, medium was decanted and cells from each 25 ml of culture were resuspended in 1 ml of structure probing buffer [50 mM HEPES-KOH pH7.9; 100 mM NaCl; 3 mM KCl]. DMS was diluted 1:6 in 100% ethanol to a final concentration of 1.76 M. Diluted DMS was added to bacteria to a final concentration of ∼105 mM. Samples were incubated with moderate shaking (800 RPM) at 25°C for 2 min, after which reactions were immediately transferred to ice. DTT was added to a final concentration of 0.7 M to quench DMS, and samples were vigorously vortexed for 10 s. Bacteria were then pelleted by centrifugation at 10 000*g* for 30 s (4°C), and the supernatant was decanted. Pellets were then washed once with 1 ml Isoamyl alcohol (Sigma–Aldrich, cat. W205702) to remove traces of water-insoluble DMS. Bacteria were then pelleted by additional centrifugation at 10 000*g* for 30 s (4°C), supernatant was decanted and samples were snap-frozen in liquid nitrogen. Pellets were stored at −80°C.

### Nucleoid isolation

Each bacteria pellet from a 25 ml culture (OD_600_ ∼0.3) was homogeneously resuspended in 200 μl of buffer A [10 mM Tris pH 8.0; 20% Sucrose; 100 mM NaCl] supplemented with 200 U SUPERase• In™ RNase Inhibitor, by pipetting. A total of 50 μl of buffer B [50 mM ethylenediaminetetraacetic acid (EDTA); 120 mM Tris pH 8.0] supplemented with 1 μl Ready-Lyse™ Lysozyme Solution (Epicentre, cat. R1810M) were added dropwise, and the vial was gently tilted five times to ensure homogenous mixing. The sample was then incubated 1 min at room temperature. A total of 250 μl of buffer C [0.5% Tween-20: 0.4% NaDOC; 2 M NaCl; 10 mM EDTA] were immediately added dropwise. The sample was then incubated 5 min at room temperature. At this stage, the solution clears considerably without increasing its viscosity, and nucleoid becomes visible. Using a cut P1000 pipette tip, the whole sample was gently layered on the top of a 5–30% w/v sucrose gradient [10 mM Tris pH 8.0; 1 M NaCl; 1 mM EDTA; 1 mM DTT] and centrifuged at 17 000 Rounds Per Minute (RPM) in a SW55Ti rotor (Beckman Coulter, cat. 342194) for 9 min (4°C). After centrifugation, the nucleoid fraction was collected using a syringe with a 18G blunt fill needle, and transferred to a new centrifuge tube. The remaining gradient was assumed to represent the cytosolic fraction. The nucleoid was then resuspended in 2.5 ml Wash & Resuspension buffer [40 mM Tris pH 7.5; 150 mM KCl; 10 mM MgCl_2_; 1 mM DTT; 0.01% Triton X-100] supplemented with 200 U SUPERase• In™ RNase Inhibitor, pulse vortexed for 5 s and then centrifuged at 28 000 RPM in a SW55Ti rotor for 30 min (0°C). After centrifugation the supernatant was decanted, and the nucleoid pellet was washed twice with 2 ml of Wash & Resuspension buffer, taking care not to disturb it. The nucleoid was then resuspended in 500 μl Wash & Resuspension buffer, and solubilized by addition of 0.1 gr acid-washed glass beads (Sigma, cat. G1145), and shaking for 5 min in a TissueLyser (QIAGEN). For each 100 μl of purified nucleoids (or cytosolic fraction), 1 ml of TRIzol^®^ Reagent (Invitrogen, cat. 15596–018) was added and RNA was extracted following manufacturer's instructions. RNA was analyzed on a 2100 Bioanalyzer (Agilent). In all experiments, RNA from cytosolic fraction (corresponding to mature RNA species) had RIN > 9.5. Total RNA yield from nucleoid fraction was ∼6% of the total RNA content.

### *In vitro* transcription from purified nucleoids

A 50 μl aliquot of solubilized nucleoid fraction was pre-equilibrated at 37°C for 5 min, then *in vitro* transcription was initiated by addition of 1 μl of NTPs (25 mM each) and incubated at 37°C for 20 min. A parallel reaction was performed without adding NTPs. Reactions were stopped by addition of 1 ml TRIzol^®^ Reagent. RNA was extracted following manufacturer's instructions, and analyzed on a 2100 Bioanalyzer.

### 3′-end RNA-seq

A total of 10 pmol of a pre-adenylated (rApp) adapter (Supplementary Table S2) were ligated to 1 μg of nascent (or mature) RNA in a reaction volume of 20 μl, using 400 U T4 RNA Ligase 2, Deletion Mutant (Epicentre, cat. LR2D11310K) in the presence of 20% PEG-8000, by incubation at 25°C for 2 h. Reaction clean-up was performed using RNA Clean & Concentrator™-5 columns, and RNA was eluted in 20 μl of Fragmentation buffer [65 mM Tris pH 8.3; 100 mM KCl; 5 mM MgCl_2_]. RNA was fragmented by incubation at 95°C for 8 min. Fragmented RNA was purified using RNA Clean & Concentrator™-5 columns, and eluted in 5.5 μl of nuclease-free water. RNA was heat-denatured at 70°C for 5 min, and reverse transcription (RT) was carried out in a final volume of 10 μl, in the presence of 0.5 mM dNTPs, 5 pmol of RT primer, 20 U RNaseOUT™ Recombinant Ribonuclease Inhibitor (Invitrogen, cat. 10777–019) and 100 U SuperScript^®^ III Reverse Transcriptase (Invitrogen, cat. 18080–044), by incubation at 50°C for 50 min. Template RNA was degraded by adding 1 μl of 1 M NaOH, and incubating at 95°C for 5 min. Reaction clean-up was performed using RNA Clean & Concentrator™-5 columns, and cDNA was eluted in 6 μl nuclease-free water. cDNA fragments were resolved on a 10% TBE-Urea polyacrylamide gel, and a gel slice corresponding to fragments in the range 40–150 nt was cut. DNA was recovered by passive diffusion in Diffusion buffer for 16 h at 37°C with moderate shaking. cDNA was precipitated by addition of 1 ml Isopropanol, and 2 μl Glycogen (20 μg/μl), and resuspended in 8.25 μl nuclease-free water. A total of 10 pmol of a 5′-phosphorylated adapter were ligated to the 3′-OH of cDNA fragments in a final reaction volume of 25 μl, in the presence of 0.05 mM ATP, 20% PEG-4000 and 100 U CircLigase™ II ssDNA Ligase (Epicentre, cat. CL9025K), by incubation at 60°C for 4 h and 68°C for 2 h. Adapter-ligated cDNA fragments were purified from excess adapter using 1.8 volumes of Agencourt AMPure XP beads (Beckman Coulter, cat. A63881), following manufacturer’s instructions. cDNA was eluted in 20 μl of nuclease-free water, and indexed sequencing adapters were introduced by 15 cycles of PCR in the presence of 25 pmol of each primer, and 25 μl NEBNext^®^ High-Fidelity 2× PCR Master Mix (NEB, cat. M0541L).

### SPET-seq library preparation of nascent RNA

A total of 10 pmol of a pre-adenylated (rApp) adapter were ligated to 1 μg of nascent RNA (either total, or rRNA-depleted using Ribo-Zero rRNA Removal Kit (Illumina, cat. MRZB12424)) in a reaction volume of 20 μl, using 400 U T4 RNA Ligase 2, Deletion Mutant in the presence of 20% PEG-8000, by incubation at 25°C for 2 h. Reaction clean-up was performed using RNA Clean & Concentrator™-5 columns, and RNA was eluted in 5.5 μl nuclease-free water. RNA was heat-denatured at 70°C for 5 min, and RT was carried out in a final volume of 10 μl, in the presence of 0.5 mM dNTPs, 5 pmol of RT primer, 20 U RNaseOUT™ Recombinant Ribonuclease Inhibitor and 100 U SuperScript^®^ III Reverse Transcriptase, by incubation at 50°C for 50 min. Template RNA was degraded by adding 1 μl of 1 M NaOH, and incubating at 95°C for 5 min. Reaction clean-up was performed using RNA Clean & Concentrator™-5 columns, and cDNA was eluted in 6 μl nuclease-free water. cDNA fragments were resolved on a 10% TBE-Urea polyacrylamide gel, and three gel slices corresponding to fragments in the ranges of 40–200, 200–400 and 400–600 nt were cut. DNA was recovered by passive diffusion in diffusion buffer for 16 h at 37°C with moderate shaking. cDNA was precipitated by addition of 1 ml Isopropanol, and 2 μl Glycogen (20 μg/μl), and resuspended in 8.25 μl nuclease-free water. A total of 10 pmol of a 5′-phosphorilated adapter were ligated to the 3′-OH of cDNA fragments in a final reaction volume of 25 μl, in the presence of 0.05 mM ATP, 20% PEG-4000 and 100 U CircLigase™ II ssDNA Ligase, by incubation at 60°C for 4 h and 68°C for 2 h. Adapter-ligated cDNA fragments were purified from excess adapter using 1.8 volumes of Agencourt AMPure XP beads, following manufacturer’s instructions. cDNA was eluted in 20 μl of nuclease-free water, and indexed sequencing adapters were introduced by 15 cycles of PCR in the presence of 25 pmol of each primer, and 25 μl NEBNext^®^ High-Fidelity 2× PCR Master Mix.

### DMS-seq library preparation of mature RNA

A total of 2 μg of RNA from the cytoplasmic fraction were diluted in 20 μl of fragmentation buffer, and fragmented by incubation at 95°C for 5 min. Fragmented RNA was purified using RNA Clean & Concentrator™-5 columns. End repair of RNA fragments was performed in a final volume of 20 μl, in the presence of 20 U T4 Polynucleotide Kinase (NEB, cat. M0201L) and 20 U SUPERase• In™ RNase Inhibitor, by incubation at 37°C for 1 h. End-repaired RNA was purified again using RNA Clean & Concentrator™-5 columns, and eluted in 6 μl of nuclease-free water. A total of 6 μl of 2× RNA Loading Dye were added to end-repaired RNA. RNA was heated to 95°C for 2 min, and immediately placed on ice. Samples were resolved on a 10% TBE-Urea polyacrylamide gel, and a gel slice corresponding to fragments above 200 nt was cut. The gel slice was crushed by centrifugation through a punctured 0.5 ml tube, and resuspended in 500 μl of diffusion buffer supplemented with 60 U SUPERase• In™ RNase Inhibitor, then rotated at 4°C for 16 h to allow passive diffusion of RNA fragments into buffer. RNA was precipitated by addition of 1 ml Isopropanol, and 2 μl Glycogen (20 μg/μl), and resuspended in 6 μl nuclease-free water. A total of 10 pmol of a pre-adenylated (rApp) adapter were ligated to size-selected RNA fragments in a reaction volume of 20 μl, using 400 U T4 RNA Ligase 2, Deletion Mutant in the presence of 20% PEG-8000, by incubation at 25°C for 2 h. Reaction clean-up was performed using RNA Clean & Concentrator™-5 columns, and RNA was eluted in 5.5 μl nuclease-free water. RNA was heat-denatured at 70°C for 5 min, and RT was carried out in a final volume of 10 μl, in the presence of 0.5 mM dNTPs, 5 pmol of RT primer, 20 U RNaseOUT™ Recombinant Ribonuclease Inhibitor and 100 U SuperScript^®^ III Reverse Transcriptase, by incubation at 50°C for 50 min. Template RNA was degraded by adding 1 μl of 1 M NaOH, and incubating at 95°C for 5 min. Reaction clean-up was performed using RNA Clean & Concentrator™-5 columns, and cDNA was eluted in 6 μl nuclease-free water. cDNA fragments were resolved on a 10% TBE-Urea polyacrylamide gel, and a gel slice corresponding to fragments in the range of 40–150 nt was cut (corresponding to truncated cDNA products). DNA was recovered by passive diffusion in diffusion buffer for 16 h at 37°C with moderate shaking. cDNA was precipitated by addition of 1 ml Isopropanol, and 2 μl Glycogen (20 μg/μl), and resuspended in 8.25 μl nuclease-free water. A total of 10 pmol of a 5′-phosphorilated adapter were ligated to the 3′-OH of cDNA fragments in a final reaction volume of 25 μl, in the presence of 0.05 mM ATP, 20% PEG-4000 and 100 U CircLigase™ II ssDNA Ligase, by incubation at 60°C for 4 h and 68°C for 2 h. Adapter-ligated cDNA fragments were purified from excess adapter using 1.8 volumes of Agencourt AMPure XP beads, following manufacturer's instructions. cDNA was eluted in 20 μl of nuclease-free water, and indexed sequencing adapters were introduced by 15 cycles of PCR in the presence of 25 pmol of each primer, and 25 μl NEBNext^®^ High-Fidelity 2× PCR Master Mix.

### SPET-seq and DMS-seq data analysis

FastQ files were examined using the FastQC tool. All the relevant SPET-seq data (nascent RNA) analysis and normalization steps were performed using a custom wrapper built on top of the RNA Framework (11). Briefly, reads were clipped from 3′ adapter sequences using Cutadapt v1.10, discarding reads shorter than 15 nt. *E. coli* str. K-12 substr. MG1655 (GenBank: U00096.2) was used as the reference genome to extract transcripts’ sequences. Forward and reverse reads were independently mapped to the reference transcriptome using Bowtie v1.1.2, by allowing up to seven mapping positions to enable mapping to the seven *E. coli* rRNA genes (parameters: -n 2 -m 7 -a –best –strata -5 5 [–norc for forward reads, –nofw for reverse reads]). Forward and reverse mapped reads were then re-paired. Using reverse read mapping positions (corresponding to RNA Polymerase positions along gene), forward reads were split into separate SAM files for each transcription intermediate. When analysis was performed in deciles of transcription, genes were split into 10 equally sized deciles and reads belonging to transcription intermediates falling in the same decile were pooled. SAM files were then passed to the *rf-count* tool of the RNA framework to generate RT-stop counts (RC) files. Resulting RC files were normalized using the *rf-norm* tool of the RNA Framework in 50 nt sliding windows, with a 25 nt offset (parameters: -sm 2 -nm 2 -ec 0 -mc 0 -n 50 -nw 50 -wo 25). Mapping of DMS-seq data (mature RNA) was performed by using the *rf-count* tool (parameters: -cl 15 -bm 7 -ba -b5 5). Resulting RC files were normalized using the *rf-norm* tool (parameters: -sm 2 -nm 2 -ec 50 -mc 50 -n 50 -nw 50 -wo 25). The *rf-norm* tool generates a XML file for each transcript (or for each transcription intermediate/decile in the case of SPET-seq data). XML files for mature RNA were passed to the *rf-fold* tool of the RNA Framework (using ViennaRNA Package 2.2 with soft constraints (12)) to infer mature RNA structures (parameters: -md 600 -nlp).

### Windowed analysis of transcription deciles

To identify regions undergoing structural changes, a dynamically sized window was sled along RNAs to identify windows containing 40 A/C residues, with a dynamic offset of 20 A/C residues. Regions with median coverage < 100 reads, and <1 RT-stop on average per A/C base were discarded. To compare consecutive deciles, windows falling >200 nt before decile’s start or within 25 nt from decile’s end were discarded to avoid biases due to lowly covered regions. This yielded a final list of ∼34 000 windows. Each window was then split into 2 × 20 nt chunks, that were independently normalized by 90% Winsorising (the value of each residue was scaled to the value of the 95th percentile). This half-normalization approach allowed smoothing the effect of coverage decrease toward the 5′-end. A Pearson coefficient cutoff of 0.8 was used to identify windows undergoing structural rearrangements.

### Deposited data

Sequencing data have been deposited on the NCBI Gene Expression Omnibus under accession GSE95567.

## RESULTS

### *In vitro* SPET-seq captures the folding pathway of RNase P

In a recent report, a method named cotranscriptional SHAPE-seq has been proposed to follow cotranscriptionally the *in vitro* folding pathway of a fluoride riboswitch (13). Although powerful, the feasibility of this approach, and the possibility to apply it to the study of *in vivo* folding, is limited by the need to produce a library of template DNA fragments to direct the synthesis of each possible RNA intermediate. To address this limitation, we developed a method named SPET-seq that exploits the fast-acting reagent DMS to modify unpaired A and C residues (14), and the stochastic distribution of RNA Polymerase along the template DNA to obtain a single-base resolution map of individual transcription intermediates. DMS has been widely employed by several recently published high-throughput structure mapping methods, and proven to be extremely effective in capturing known RNA structures. Moreover, DMS is a fast-acting reagent, thus making it more suitable to capture the transient structure of transcription intermediates compared to slow-acting reagents such as NMIA, NAI, CMCT and Kethoxal (15–17). SPET-seq simultaneously captures both the 3′-end of the nascent transcript (RNA Polymerase position), and the site of DMS modification, thus allowing to visualize the secondary structure of individual transcription intermediates in a single experiment.

**Figure 1.**
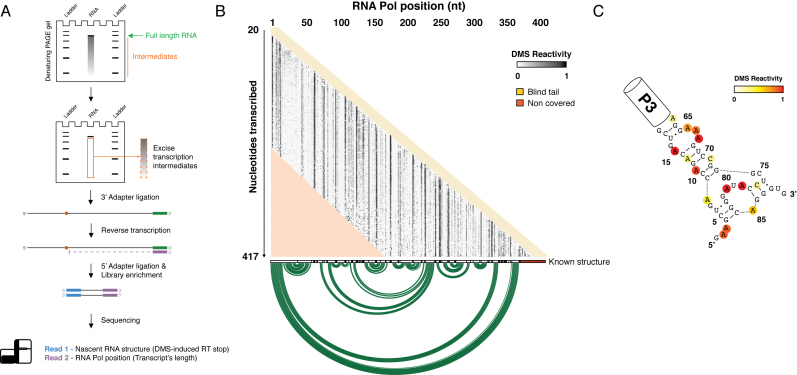
Overview of *in vitro* SPET-seq. (**A**) Outline of *in vitro* SPET-seq method applied to RNase P gene (rnpB). (**B**) Heatmap of per-base DMS reactivity across rnpB transcription intermediates. Regions marked in yellow and red respectively represent the blind region due to minimum read length required for mapping, and the non-covered region of the transcription intermediate. (**C**) SPET-seq reactivity data overlaid on transient non-native structure previously observed by Wong *et al.*, (10).

To validate the method, we performed *in vitro* transcription of *Escherichia coli* RNase P RNA (rnpB). RNase P is a ribozyme essential for the maturation of tRNAs and several other non-coding RNAs (18). In our assay, the rnpB gene along with its natural promoter and Rho-independent terminator (Supplementary Figure S1) was transcribed using a sigma70-saturated *E. coli* RNA Pol holoenzyme (Figure 1A). Transcription was allowed to proceed for 5 min, after which elongation by RNA Pol was halted by the simultaneous addition of Actinomycin D and DNase I. RNA was then treated with DMS, and resolved on a polyacrylamide gel to recover transcription intermediates. To directly capture the length of individual transcription intermediates, RNA 3′-ends were ligated to a pre-adenylated adapter, that was used to drive RT. Single-stranded residues were detected as the nucleotide immediately downstream of the RT-stop induced by the DMS modification. A second phosphorylated adapter was then ligated to the 3′-end of cDNA molecules, and the library was enriched by PCR. After performing paired-end sequencing, forward reads were split according to mapping position of the reverse mate, thus allowing visualization of individual rnpB transcription intermediates at single-base resolution (Figure 1B). According to a previous report, co-transcriptional folding of RNase P RNA is characterized by the formation of a non-native secondary structure that sequesters the 5′ portions of long-range helices until their 3′ counterparts have been transcribed (10). Concordantly, base reactivities measured by SPET-seq were in strong agreement with the previously proposed structure of the non-native intermediate (Figure 1C).

Together these data demonstrate the suitability of SPET-seq for the study of cotranscriptional RNA folding.

### *In vivo* isolation of transcription intermediates

Given the positive results obtained *in vitro*, we decided to apply SPET-seq *in vivo*. We chose *E. coli* as our model organism for two main reasons. First, *E. coli* genes lack introns, thus lowering the sequencing depth required to obtain sufficient structural information on exons. Second, *E. coli* genes are mostly very short, with a median length of about 800 bp (see Supplementary Note S1).

We then sought to develop a method for the rapid isolation of nascent RNA. *E. coli* genome consists of a circular DNA molecule that is packed into a compact body, known as *nucleoid* (19). Previous reports demonstrated that the nucleoid can be isolated in a compact conformation, retaining almost uniquely elongating RNA Pol and nascent RNA chains (20,21). To evaluate the suitability of this approach for isolating nascent RNA, we isolated nucleoid from log-phase *E. coli* (Supplementary Figure S2A). When supplied with NTPs, this DNA–RNA–RNA Pol complex resumed transcription *in vitro* (Supplementary Figure S2B). Nucleoid-associated RNA accounted for ∼6% of total cellular RNA content (Supplementary Figure S2C). To characterize nucleoid-associated RNA, we performed 3′-end RNA-seq (Supplementary Figure S2D) of both nucleoid and cytosolic fractions. To this end, we directly ligated to RNA the pre-adenylated Illumina 5′ adapter and used a complementary oligonucleotide to drive RT. Analysis of this data showed that, while 3′-end cytosolic RNA signal was almost exclusively enriched toward transcription end sites, nucleoid RNA signal accumulated along gene bodies (Supplementary Figure S2E). We were also able to detect known RNA Pol pause sites (22,23) (Supplementary Figure S2F). Additionally, collapsing of duplicate reads provided an overview of RNA Pol occupancy along *E. coli* genome, that well agreed with a previously published RNA Pol ChIP-chip dataset (24) (Supplementary Figure S2G). Moreover, nucleoid-associated RNA extraction was extremely reproducible (*R* = 0.99, Supplementary Figure S2H). Altogether, these data suggested that nucleoid extraction is a straightforward and accurate approach for nascent RNA isolation.

### *In vivo* SPET-seq reveals principles of cotranscriptional RNA structure formation

**Figure 2.**
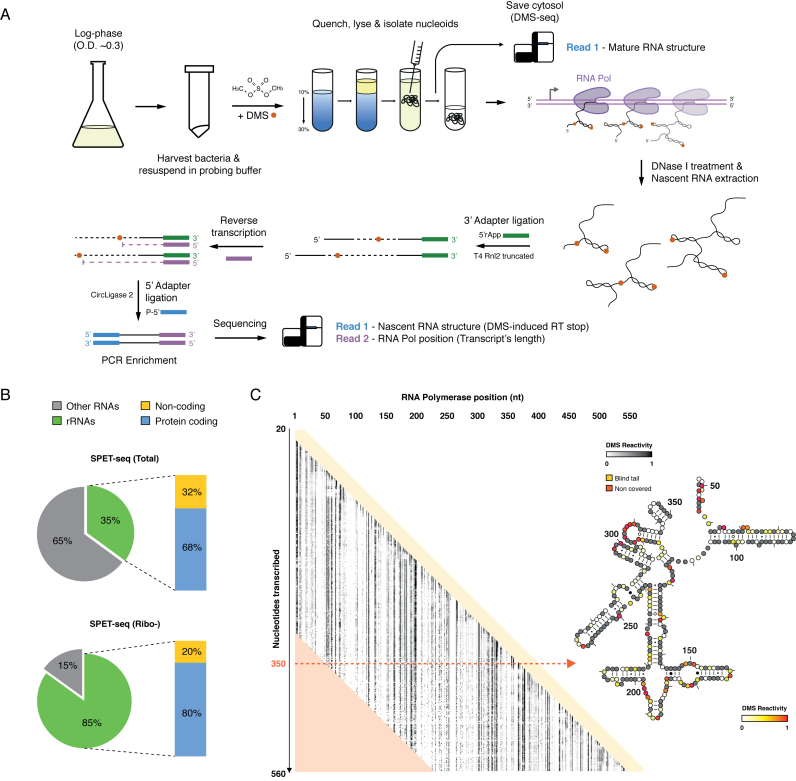
SPET-seq captures RNA folding intermediates *in vivo*. (**A**) Outline of *in vivo* SPET-seq applied to *Escherichia coli*. (**B**) Distribution of read mappings for *in vivo* SPET-seq on total and ribo-depleted nascent RNA. (**C**) Heatmap of per-base DMS reactivity across transcription intermediates in the 5′-terminal domain of 16S rRNA (rrsB).

To perform *in vivo* SPET-seq, *E. coli* grown to log-phase were treated with DMS to modify single-stranded RNA residues (Figure 2A). Nucleoid fraction was isolated, and used for SPET-seq library preparation. In parallel, cytosolic fraction was saved and used for DMS-seq library preparation (25) to obtain a snapshot of mature RNA structures. We sequenced around 700 million paired-end reads, and obtained single-base resolution structural information for transcription intermediates of both ribosomal RNAs and mRNAs (Figure 2B and C; Supplementary Figures S3 and 4).

**Figure 3.**
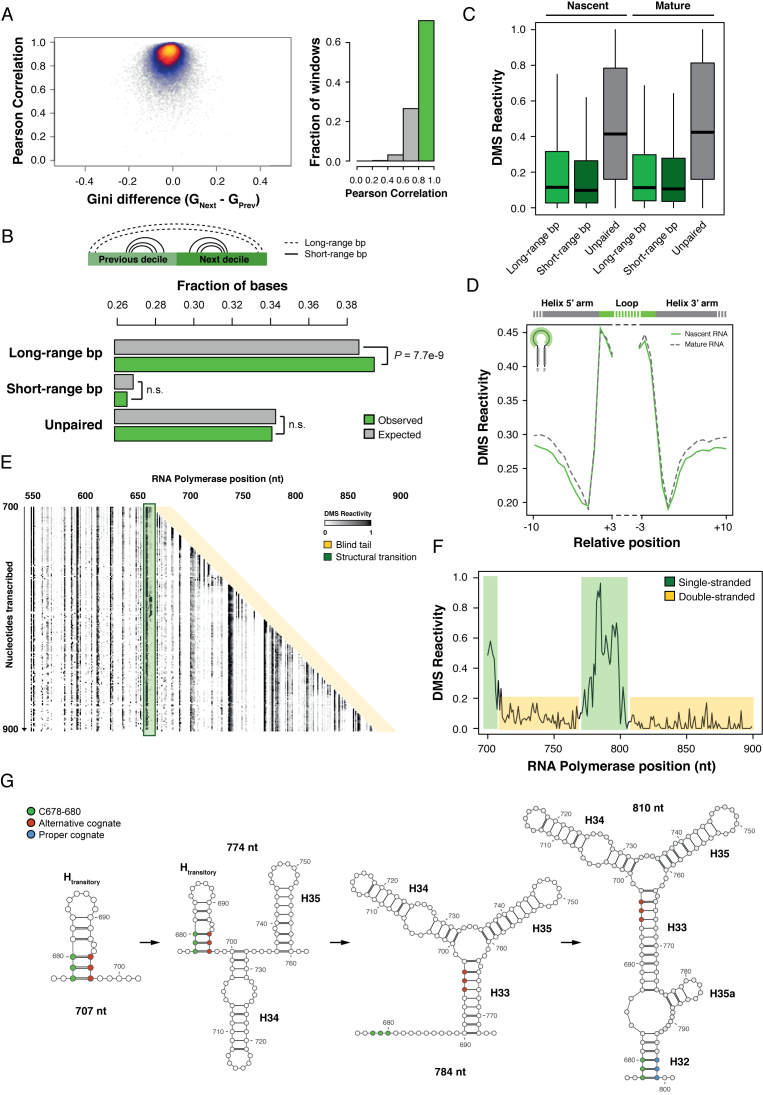
RNA cotranscriptional folding *in vivo*. (**A**) (Left) Scatter-plot of Gini difference versus Pearson Correlation for ∼34 000 windows of 40 A/C residues in consecutive deciles (Prev and Next). (Right) Distribution of Pearson Correlation coefficients across analyzed windows. (**B**) Bar-plot of expected versus observed fraction of unpaired bases, or bases involved in either long/short-range interactions within windows with *r* < 0.8. (**C**) Box-plot of DMS reactivities for bases which are either unpaired or involved in long/short-range base pairs in nascent and mature RNA. (**D**) Average normalized reactivity on minimal RNA secondary structure elements (individual hairpins, short-range interactions) in the mature RNA structure (dashed line). Nascent RNA signal (solid line) is derived from the decile in which the structural element has been transcribed. (**E**) Heatmap of per-base DMS reactivity across 23S rRNA (rrlB) transcription intermediates in a region comprised between position 700 and 900. Green window highlights C678-C680. (**F**) Average DMS reactivity trajectory for residues C678-C680. (**G**) Structural transitions observed during the cotranscriptional folding of helix 23 of 23S rRNA.

To systematically characterize the cotranscriptional folding dynamics of the *E. coli* transcriptome, we divided genes into 10 equally-sized segments (deciles), and using a sliding-window approach we compared ∼34 000 windows of 40 A/C residues across consecutive transcription deciles using two previously defined metrics, the Pearson correlation coefficient and the Gini index difference (25) (Figure 3A). Surprisingly, around 71% of analyzed windows showed both high correlation (*r* > 0.8) across transcription stages, and negligible Gini index difference (|Gini difference| < 0.1), suggesting that most structures formed during transcription are stable. We next focused on 29% of windows undergoing structural rearrangements (Supplementary Table S1). For these windows, we did not observe any difference between those belonging to protein-coding or non-coding transcripts (Supplementary Figure S5A), or in their GC content (Supplementary Figure S5B). Instead, we observed that these windows were enriched for genes involved in metabolic and biosynthetic processes (Supplementary Figure S6).

We then used DMS-seq data from cytosolic fraction to perform whole transcriptome soft constraint-guided inference of mature RNA secondary structures (11,12). RNA bases were split into three categories: bases involved in short-range interactions (when both bases involved in a base-pair are transcribed within the same transcription decile), long-range interactions (when the base-pair spans across two transcription deciles) and unpaired bases (see ‘Materials and Methods’ section). Notably, windows undergoing structural changes were enriched for residues involved in long-range interactions (*P* = 7.7e-9, Hypergeometric distribution; Figure 3B). We next examined the reactivity to DMS of unpaired bases, and of bases residing in the 5′-half of both short and long-interaction helices, within the respective transcription decile compared to the mature RNA (Figure 3C). Bases involved in the formation of short-range interactions (individual hairpins) exhibited the same distribution of reactivities in both nascent and mature RNA. Analysis of both 5′ and 3′ halves of these short-range helices revealed that they are formed as soon as they get transcribed (Figure 3D). Also, the distribution of base reactivities observed for bases residing within the 5′-half of long-range interactions was comparable between nascent and mature RNA (*P* = 0.2, One-way ANOVA), thus suggesting that these bases get sequestered into transitory interactions until their cognates get transcribed.

As an example, by analyzing 23S rRNA folding pathway we detected a structural transition involving residues C678-C680 (Figure 3E and F). These residues are involved in the formation of helix 32 of 23S rRNA. As these residues emerge from the RNA Polymerase channel, they are characterized by high DMS reactivity, that is lost when RNA Pol reaches position 707, due to their sequestering into a transitory helix involving pairing with residues G695–G697 (Figure 3G). As position 780 gets transcribed, residues C765–U766–U767 sequester G695–G697, leaving C678–C680 unpaired until the proper cognate bases G797–G799 have been transcribed.

## DISCUSSION

In this work, we introduced a new method, named SPET-seq, that enables base-resolution analysis of transcription intermediate RNA structures. To our knowledge, this is the first report that evaluates the cotranscriptional RNA structure formation on a transcriptome-wide scale in a living organism.

After validating the suitability of SPET-seq *in vitro*, we applied this technique to *E. coli* to gain insights into *in vivo* cotranscriptional structure formation. By systematically analyzing cellular RNAs, we observed that the majority of them don’t undergo significant structural transitions along their folding pathways. Instead, around 29% of analyzed windows sample different conformations across transcription stages. This fraction probably represents a conservative estimate of the actual number of structure-changing regions, as the intrinsic limitation of SPET-seq is to allow the resolution of the 3′-most portion of nascent RNA chains only (see Supplementary Note S1). These windows did not show any significant bias toward lower GC contents, or across different RNA classes. Instead, they are enriched within genes involved in metabolic and biosynthetic processes. We can speculate that these regions might act as riboswitch-like regions to sense metabolite levels.

By using structure probing data from mature cytosolic RNAs, we inferred their structure by soft constraint-guided folding, and stratified RNA bases into unpaired, involved in short-range interactions or involved in long-range interactions. Bases involved in long-range interactions were significantly enriched within structure-changing windows. Comparison of reactivity distributions for bases residing in the 5′-halves of these long-range helices between mature RNA and nascent RNA did not show any significant difference, thus suggesting that these bases get immediately sequestered into non-native transitory interactions until their 3′ counterpart has been transcribed.

As a proof of this, we detected a structural transition involving the formation of helix 32 of 23S rRNA. By performing soft constraint-guided modeling we observed that a cluster of three C residues (C678–C680) gets sequestered by a nearby cluster of three G residues (G695–G697) to form a transitory helix. As the true cognate bases of G695–G697 get transcribed, residues C678–C680 are immediately released, and remain single-stranded until the proper 3′-cognates G797–G799 have been transcribed.

No difference was observed also for unpaired and short-range interacting bases. Notably, the same reactivity profile between mature and nascent RNA was observed at the level of local structured domains (individual hairpins), thus suggesting that these hairpins get folded as soon as they get transcribed. So, it appears that the kinetics of transcription plays a major role in regulating the formation of these structures.

**Figure 4.**
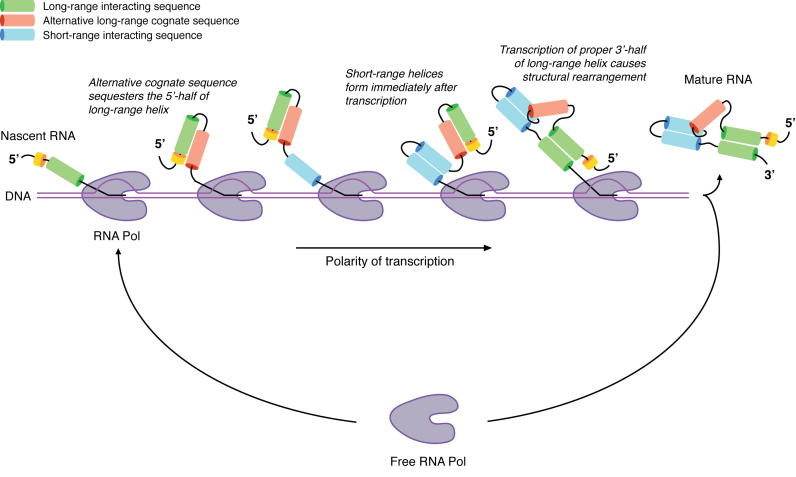
Model of *in vivo* cotranscriptional folding. Proposed model of *in vivo* RNA folding dynamics as revealed by SPET-seq analysis.

Collectively our data suggests that cotranscriptional RNA folding *in vivo* is a process in which short-range interactions are immediately formed, while long-range interactions require the formation of non-native transitory structures in order to sequester the 5′ side of long-range helices (Figure 4).

SPET-seq constitutes a solid and straightforward method for the study of RNA cotranscriptional folding pathways both *in vivo* and *in vitro*. We can anticipate that SPET-seq might be exploited to provide insights into the more complex cotranscriptional folding landscape of eukaryotic RNAs. Although nascent RNA probing of eukaryotic RNAs might be harder due to the presence of intronic sequences long up to hundreds of kilobases, an exon enrichment step based on the use of biotinylated capture probes can be easily inserted at the end of the SPET-seq library preparation procedure to enrich exonic reads.

## ACCESSION NUMBER

GSE95567.

## Supplementary Material

Supplementary Data
