# Supplementary material for: In vivo probing of nascent RNA structures reveals principles of cotranscriptional folding
Source: Nucleic Acids Res. 2017 Jul 14;45(16):9716–25. doi: 10.1093/nar/gkx617 (PMC5766169; doi:10.1093/nar/gkx617)
Supplement: Supplementary Data [file gkx617_supp.zip › nar-01601-f-2017-File006.pdf]

Note S1.

In the SPET-seq protocol, a pre-adenylated adapter is ligated to the 3'-OH end of nascent RNA molecules, and used to drive reverse transcription. Although this allows detecting RNA Polymerase position, it causes SPET-seq coverage to be skewed toward the 3'-end of RNA molecules. This is the major limitation of SPET-seq. With a median coverage of 50X, SPET-seq is able to cover ~200 nt of each RNA intermediate (Panel A), roughly corresponding to 1/4th of the median *E. coli* gene (~800 nt, Panel B).

A

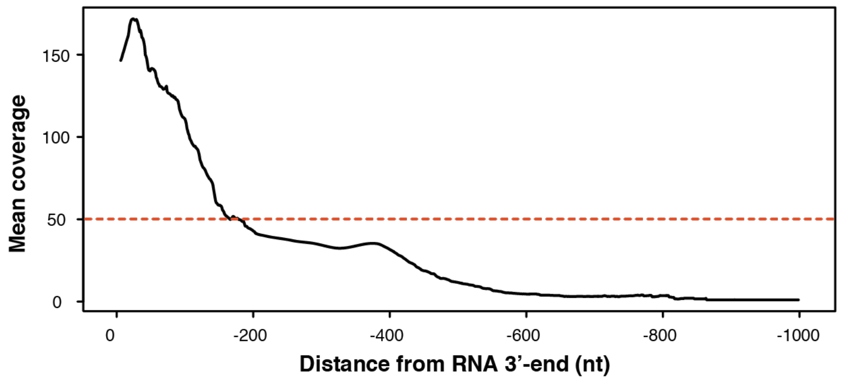

B

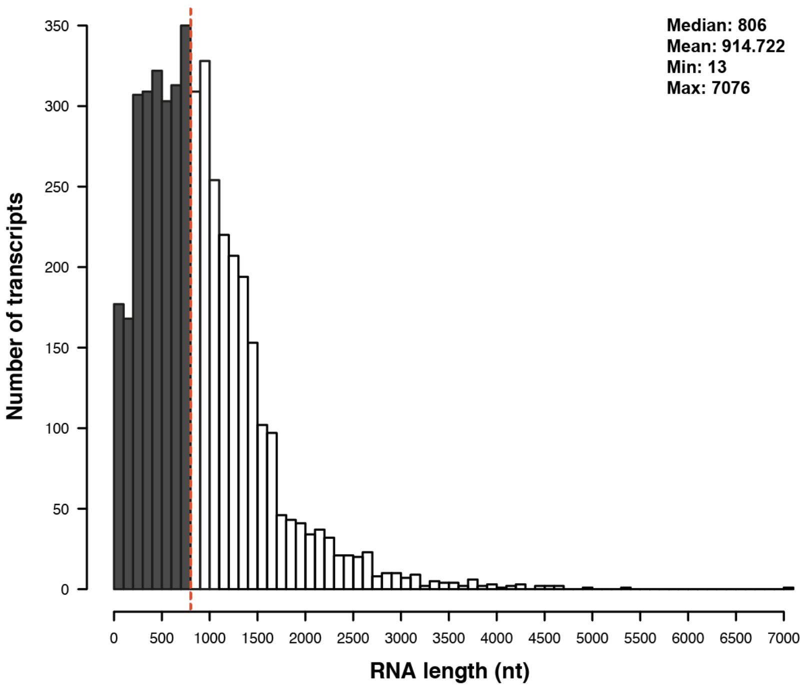

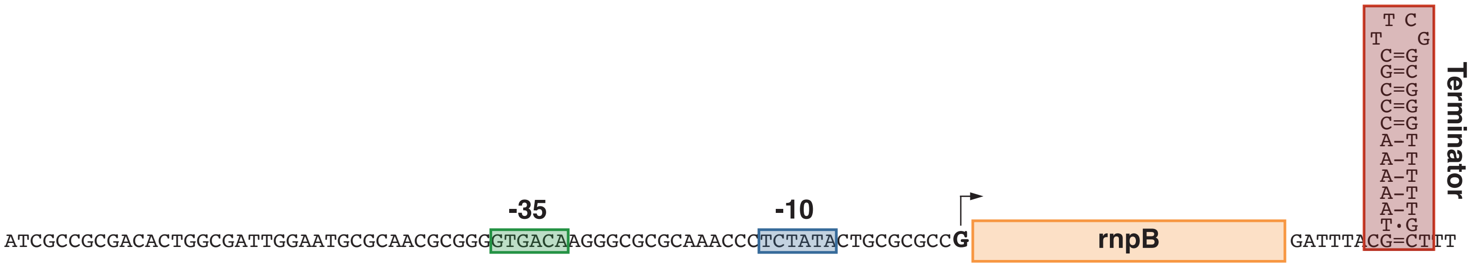

-35
-10
ATCGCCGCGACACTGGCGATTGGAATGCGCAACGCGGGGTGACAAGGGCGCGCAAACCCTCTATACTGCGCGCCG
rnpB
GATTTACG=CTTT
T C
T G
C=G
G=C
C=G
C=G
C=G
A–T
A–T
A–T
A–T
A–T
T•G
Terminator

Figure S1.

Schematic of the rnpB template used for *in vitro* SPET-seq.

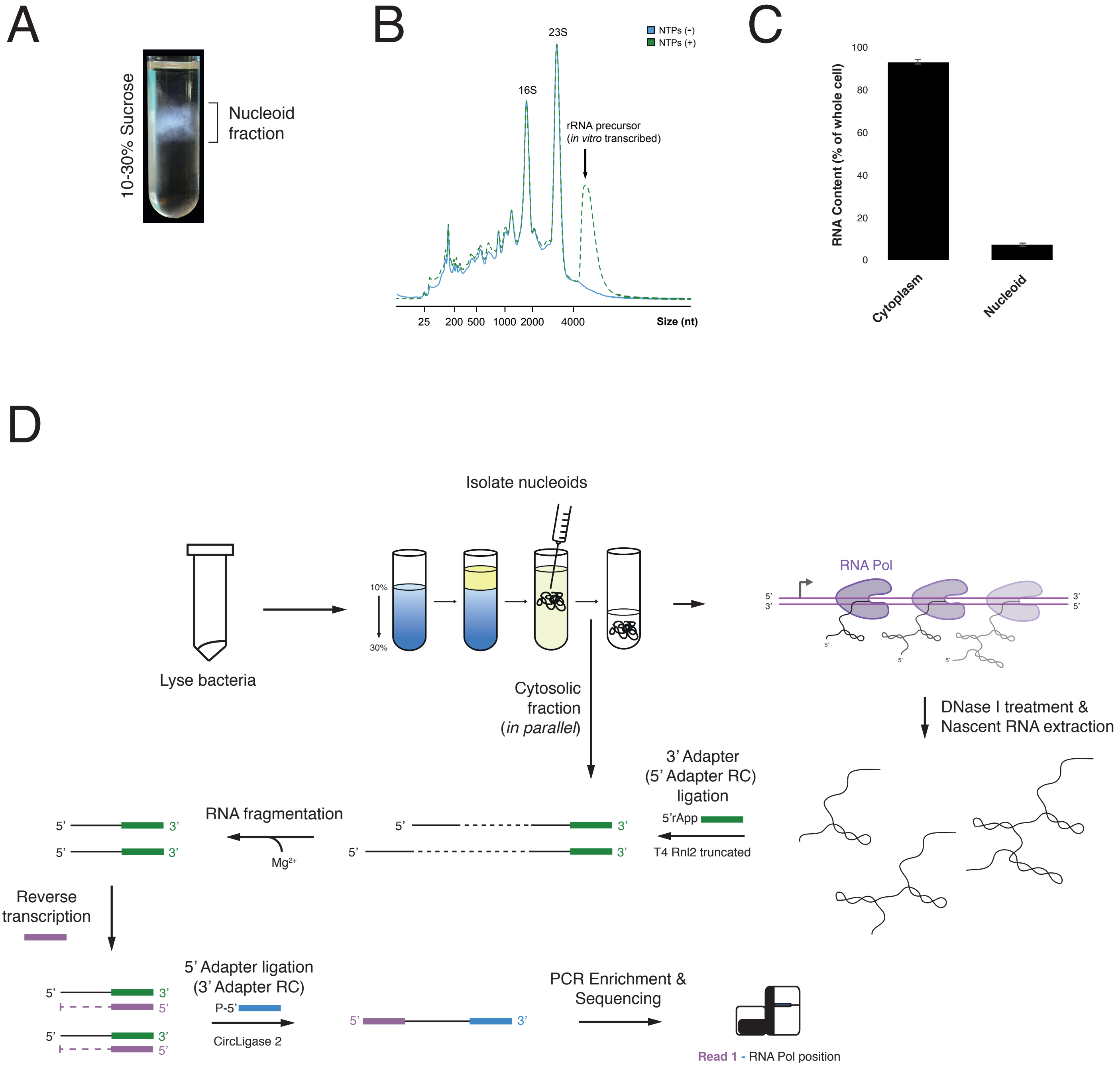
A
10-30% Sucrose
Nucleoid fraction
B
23S
16S
NTPs (-)
NTPs (+)
rRNA precursor
(in vitro transcribed)
25
200
500
1000
2000
4000
Size (nt)
C
RNA Content (% of whole cell)
100
80
60
40
20
0
Cytoplasm
Nucleoid
D
Isolate nucleoids
10%
30%
Lyse bacteria
RNA Pol
Cytosolic fraction (in parallel)
DNase I treatment & Nascent RNA extraction
3' Adapter (5' Adapter RC) ligation
5'rApp
T4 Rnl2 truncated
RNA fragmentation
Mg2+
Reverse transcription
5' Adapter ligation (3' Adapter RC)
P-5'
CircLigase 2
PCR Enrichment & Sequencing
Read 1 - RNA Pol position

E

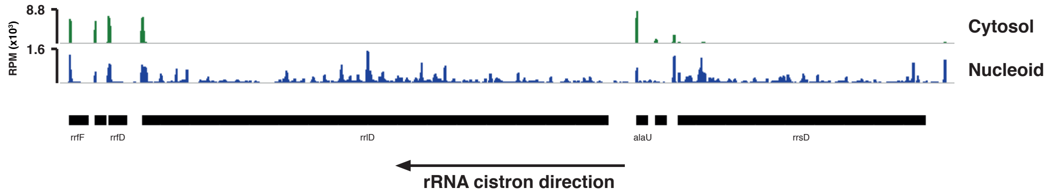

8.8
1.6
RPM (x10³)
Cytosol
Nucleoid
rrfF
rrfD
rrlD
alaU
rrsD
rRNA cistron direction

F

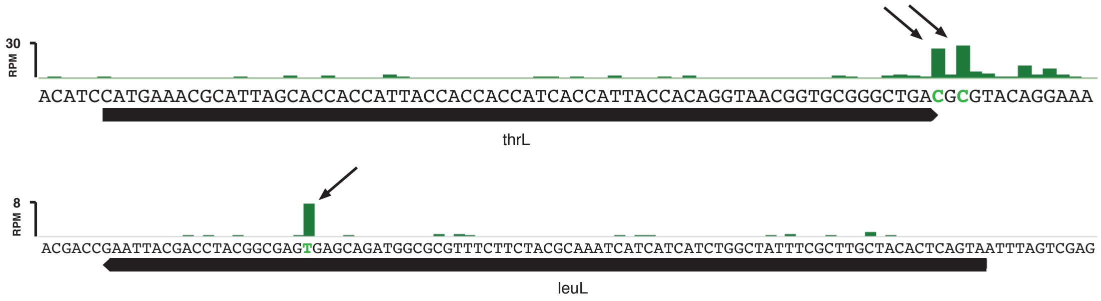

30
RPM
ACATCCATGAAACGCATTAGCACCACCATTACCACCACCATCACCATTACCACAGGTAACGGTGCGGGCTGACGCGTACAGGAAA
thrL
8
RPM
ACGACCGAATTACGACCTACGGCGAGTGAGCAGATGGCGCGTTTCTTCTACGCAAATCATCATCATCTGGCTATTTCGCTTGCTACACTCAGTAATTTAGTCGAG
leuL

G

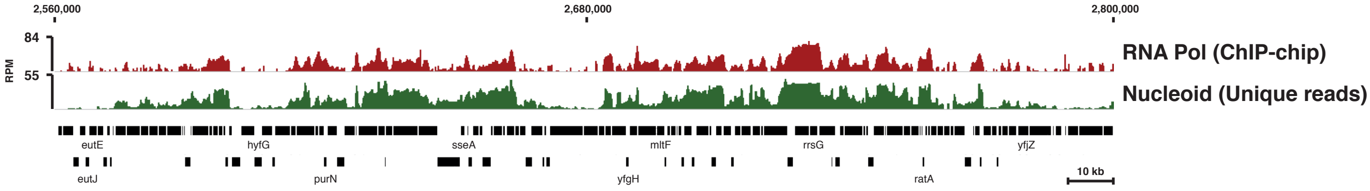

2,560,000
2,680,000
2,800,000
84
55
RPM
RNA Pol (ChIP-chip)
Nucleoid (Unique reads)
eutE
hyfG
sseA
mltF
rrsG
yfjZ
eutJ
purN
yfgH
ratA
10 kb

H

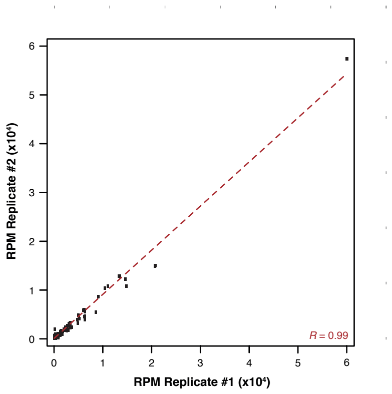

RPM Replicate #2 (x10⁴)
RPM Replicate #1 (x10⁴)
R = 0.99

Figure S2

(**A**) Photo of nucleoid fraction after centrifugation through sucrose gradient. (**B**) Bioanalyzer traces of RNA from nucleoid fractions incubated at 37°C for 20 minutes with or without NTPs (0.5 mM each). Accumulation of a transcription product > 4000 nt is clearly visible in the presence of NTPs. (**C**) RNA yields from cytosolic and nucleoid fractions. (**D**) Outline of the 3'-end RNA-seq protocol. (**E**) Genomic view of 3'-end RNA-seq signal (enlarged by 20 bp) across one rRNA cistron. (**F**) Sequencing of nucleoid-associated nascent RNA accurately captures previously identified RNA Polymerase pausing sites. (**G**) Genomic view of 3'-end RNA-seq signal after collapsing identical reads provides a map of RNA Polymerase occupancy, and is in good agreement with a previous ChIP-chip dataset (Myers *et al.*, 2013). (**H**) Nucleoid-associated RNA extraction is highly reproducible. Nascent RNA abundance at single-base resolution is highly correlated across two biological replicates (R = 0.99).

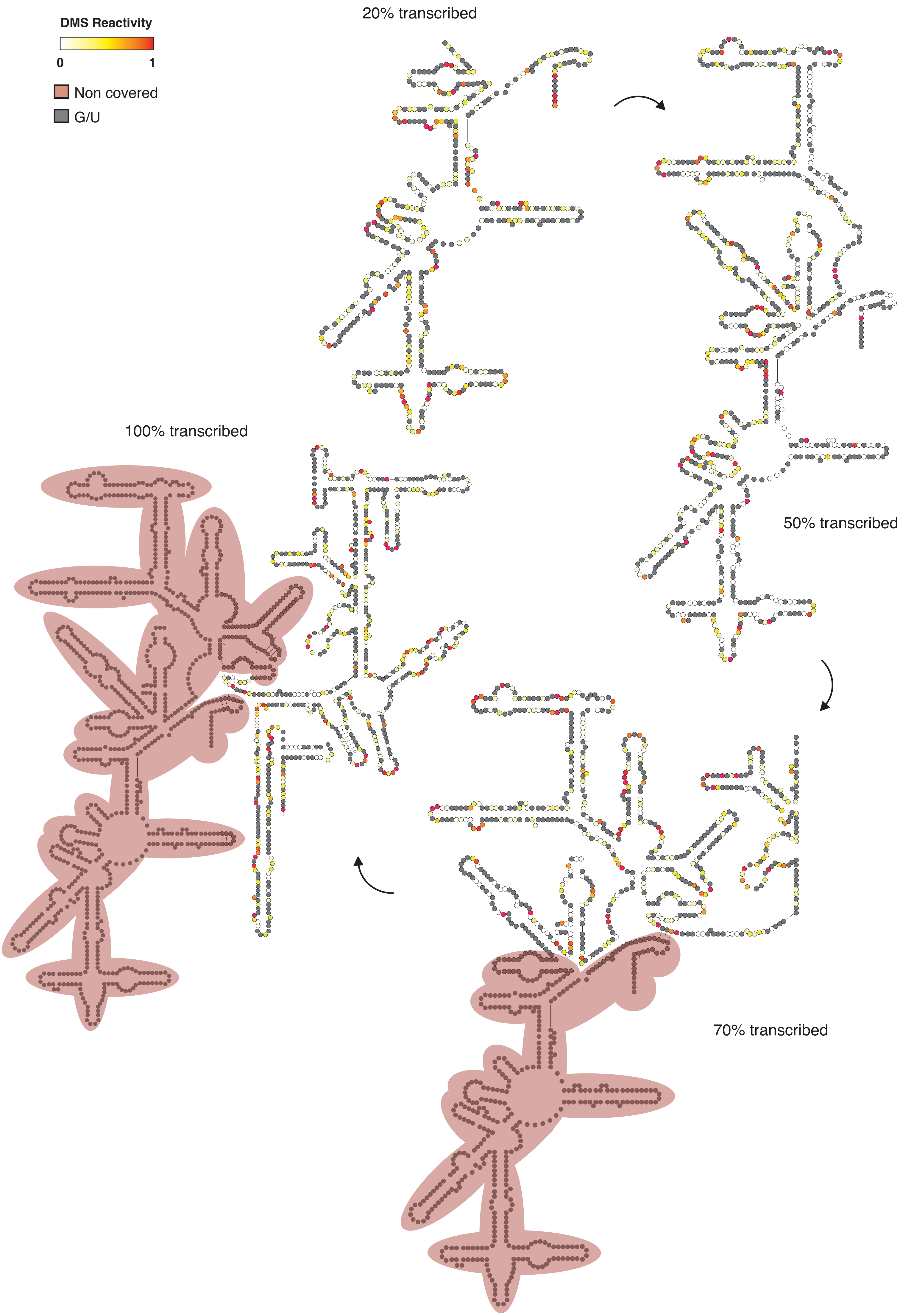
DMS Reactivity
0
1
Non covered
G/U
20% transcribed
50% transcribed
70% transcribed
100% transcribed

Figure S3

DMS reactivities across 2nd, 5th, 7th, and 10th transcription deciles overlaid on the phylogenetic-derived structure of 16S rRNA (rrsB).

A

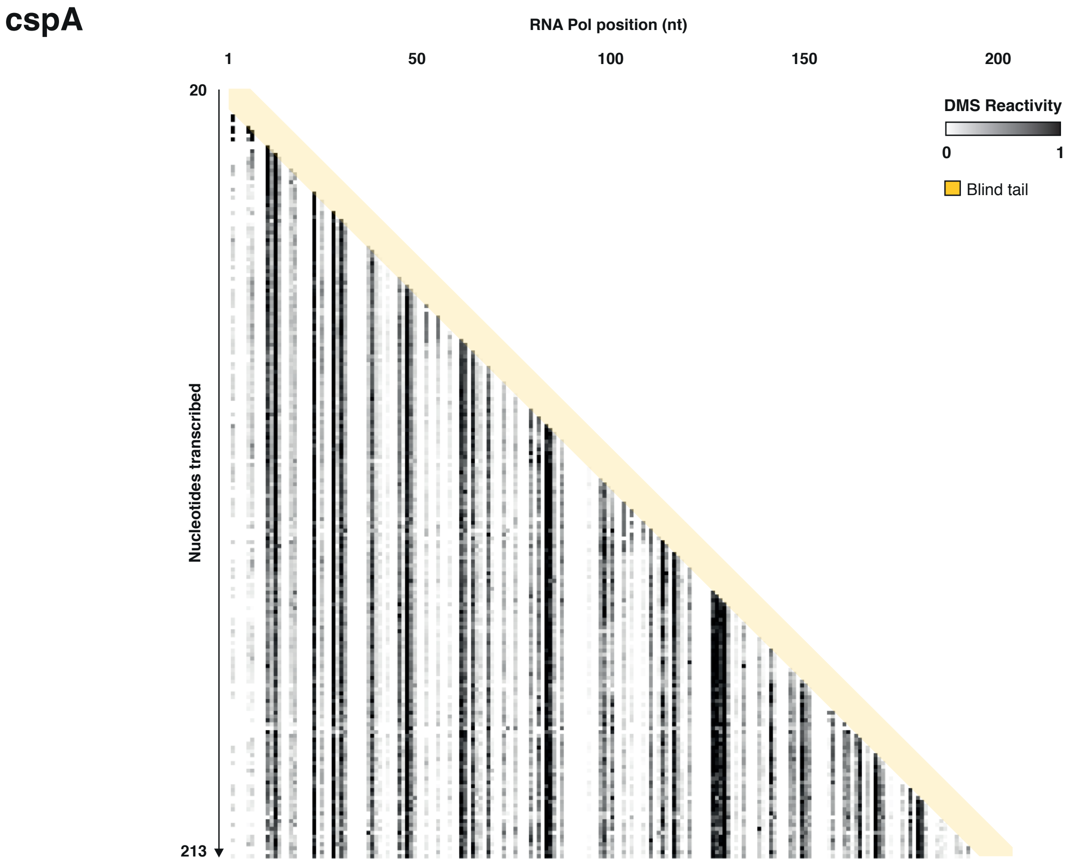
cspA
RNA Pol position (nt)
1
50
100
150
200
20
213
Nucleotides transcribed
DMS Reactivity
0
1
Blind tail

B

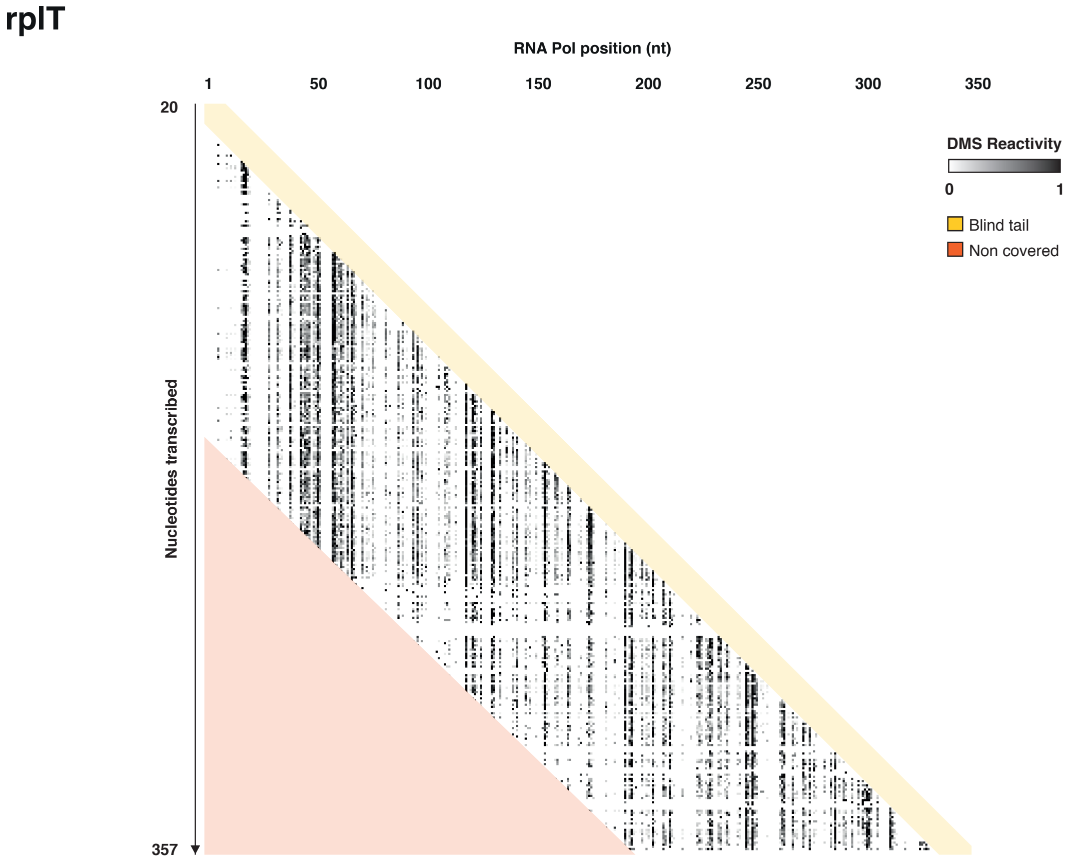
rplT
RNA Pol position (nt)
1
50
100
150
200
250
300
350
20
357
Nucleotides transcribed
DMS Reactivity
0
1
Blind tail
Non covered

Figure S4

(**A**) Heatmap of per-base DMS reactivity across cspA transcription intermediates. (**B**) Heatmap of per-base DMS reactivity across rplT transcription intermediates. Regions marked in yellow and red respectively represent the blind region due to minimum read length required for mapping, and the non-covered region of the transcription intermediate.

A

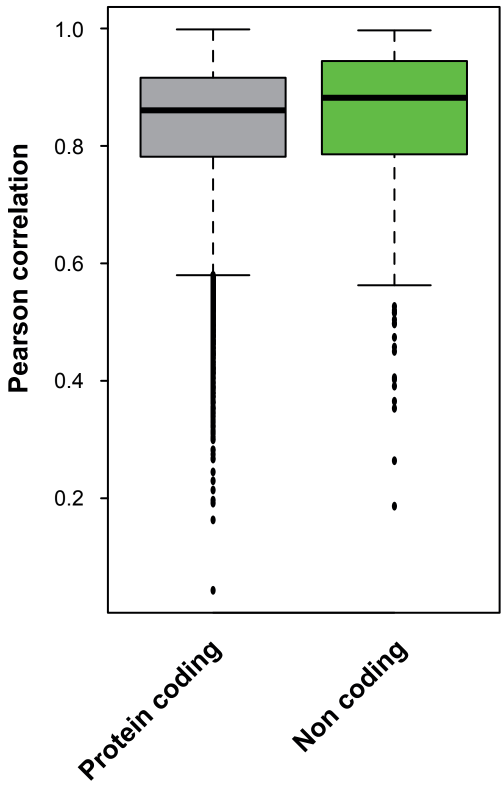
1.0
0.8
0.6
0.4
0.2
Pearson correlation
Protein coding
Non coding

B

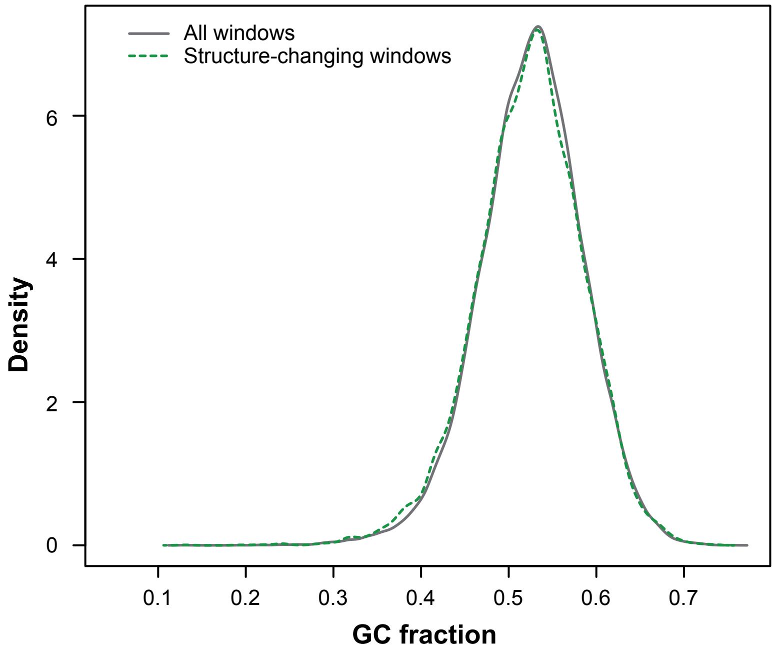
All windows
Structure-changing windows
6
4
2
0
Density
0.1
0.2
0.3
0.4
0.5
0.6
0.7
GC fraction

Figure S5

(**A**) Box-plot of Pearson correlation coefficients for analyzed windows belonging either to protein-coding or non-coding transcripts. (**B**) Density plot of fraction GC abundances inside all analyzed windows (grey solid line), compared to windows undergoing structural changes (green dashed line).

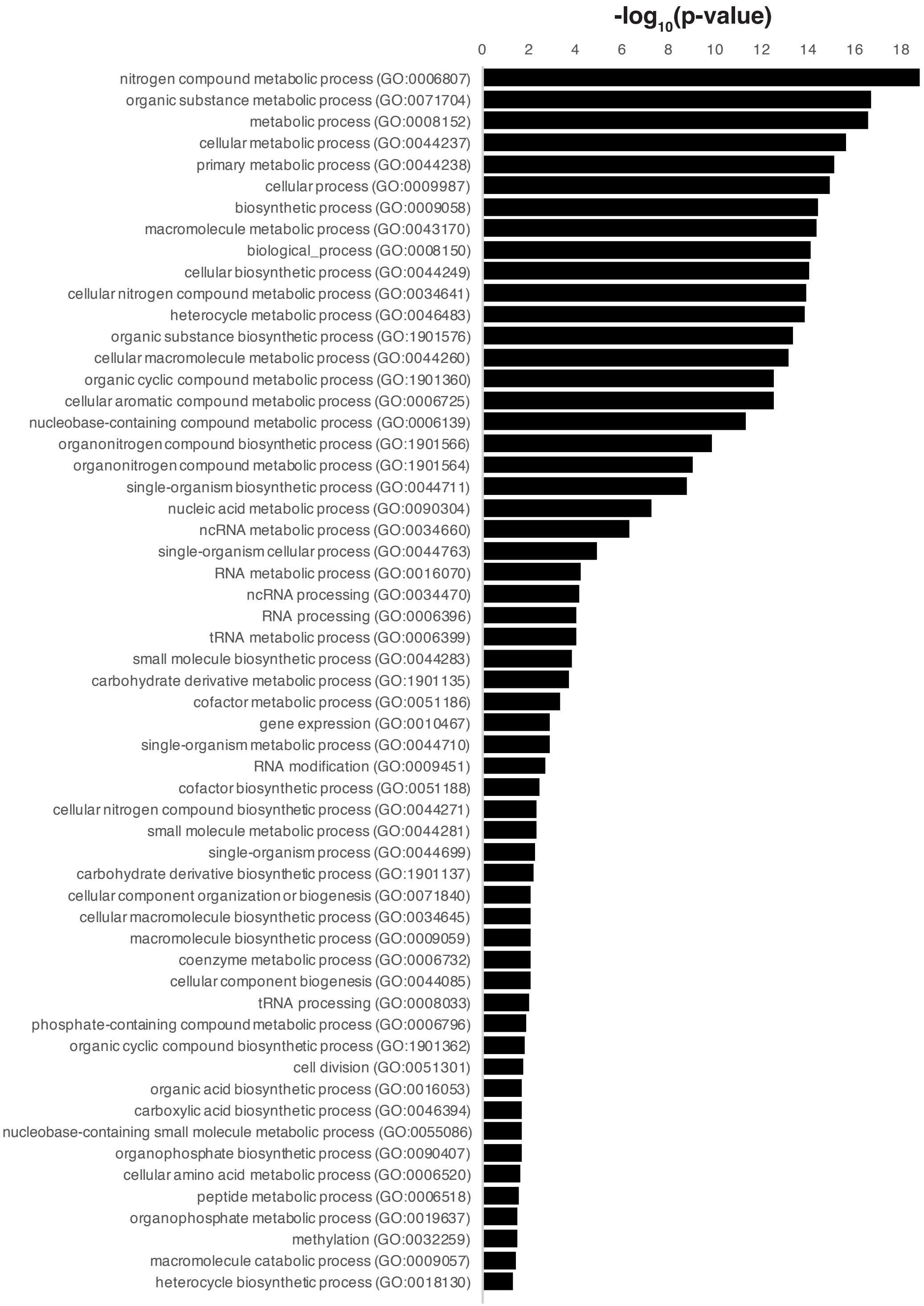

-log10(p-value)
0
2
4
6
8
10
12
14
16
18
nitrogen compound metabolic process (GO:0006807)
organic substance metabolic process (GO:0071704)
metabolic process (GO:0008152)
cellular metabolic process (GO:0044237)
primary metabolic process (GO:0044238)
cellular process (GO:0009987)
biosynthetic process (GO:0009058)
macromolecule metabolic process (GO:0043170)
biological_process (GO:0008150)
cellular biosynthetic process (GO:0044249)
cellular nitrogen compound metabolic process (GO:0034641)
heterocycle metabolic process (GO:0046483)
organic substance biosynthetic process (GO:1901576)
cellular macromolecule metabolic process (GO:0044260)
organic cyclic compound metabolic process (GO:1901360)
cellular aromatic compound metabolic process (GO:0006725)
nucleobase-containing compound metabolic process (GO:0006139)
organonitrogen compound biosynthetic process (GO:1901566)
organonitrogen compound metabolic process (GO:1901564)
single-organism biosynthetic process (GO:0044711)
nucleic acid metabolic process (GO:0090304)
ncRNA metabolic process (GO:0034660)
single-organism cellular process (GO:0044763)
RNA metabolic process (GO:0016070)
ncRNA processing (GO:0034470)
RNA processing (GO:0006396)
tRNA metabolic process (GO:0006399)
small molecule biosynthetic process (GO:0044283)
carbohydrate derivative metabolic process (GO:1901135)
cofactor metabolic process (GO:0051186)
gene expression (GO:0010467)
single-organism metabolic process (GO:0044710)
RNA modification (GO:0009451)
cofactor biosynthetic process (GO:0051188)
cellular nitrogen compound biosynthetic process (GO:0044271)
small molecule metabolic process (GO:0044281)
single-organism process (GO:0044699)
carbohydrate derivative biosynthetic process (GO:1901137)
cellular component organization or biogenesis (GO:0071840)
cellular macromolecule biosynthetic process (GO:0034645)
macromolecule biosynthetic process (GO:0009059)
coenzyme metabolic process (GO:0006732)
cellular component biogenesis (GO:0044085)
tRNA processing (GO:0008033)
phosphate-containing compound metabolic process (GO:0006796)
organic cyclic compound biosynthetic process (GO:1901362)
cell division (GO:0051301)
organic acid biosynthetic process (GO:0016053)
carboxylic acid biosynthetic process (GO:0046394)
nucleobase-containing small molecule metabolic process (GO:0055086)
organophosphate biosynthetic process (GO:0090407)
cellular amino acid metabolic process (GO:0006520)
peptide metabolic process (GO:0006518)
organophosphate metabolic process (GO:0019637)
methylation (GO:0032259)
macromolecule catabolic process (GO:0009057)
heterocycle biosynthetic process (GO:0018130)

Figure S6

Gene ontology analysis performed on genes with structure-changing windows.

Supplementary Tables

Table S1. Analyzed RNA windows undergoing structural rearrangements.

Table S2. Sequence of oligonucleotides used in this study.
